# Anti-TNBC effects of Lappaol F by targeting epithelial-mesenchymal transition via regulation of GSK-3β/YAP/β-catenin and PI3K/AKT pathways

**DOI:** 10.3389/fphar.2025.1496511

**Published:** 2025-02-07

**Authors:** Qiqi Meng, Zhiping Li, Xiaofeng He, Yuanhao Hu, Guiyun Wu, Jiawen Huang, Zhuohui Luo, Yingjie Hu, Xiaoling Shen

**Affiliations:** ^1^ Science and Technology Innovation Center, Guangzhou University of Chinese Medicine, Guangzhou, Guangdong, China; ^2^ International Institute for Translational Chinese Medicine, School of Pharmaceutical Science, Guangzhou University of Chinese Medicine, Guangzhou, Guangdong, China; ^3^ Research Center for Drug Safety Evaluation of Hainan Province, Hainan Medical University, Haikou, Hainan, China; ^4^ Hainan Pharmaceutical Research and Development Science Park, Haikou, Hainan, China

**Keywords:** Arctium lappa L, Lappaol F, triple negative breast cancer, epithelial-mesenchymal transition, GSK-3β/YAP/β-catenin signaling, PI3K/AKT signaling

## Abstract

**Purpose:**

Lappaol F (LAF), a lignan extracted from *Fructus Arctii*, has a wide spectrum of anti-tumor effects, including inhibition of TNBC cell growth. However, the pharmacological mechanism of LAF targeting epithelial-mesenchymal transition (EMT) to inhibit Triple-negative breast cancer (TNBC) growth remains poorly understood. The present study aimed to reveal the potential mechanism of LAF against TNBC by *in vivo* and *in vitro* experiments.

**Methods:**

*In situ*, transplantation-induced MDA-MB-231 solid tumor model in NCG mice and cultured MDA-MB-231 and Hs-578T cells were used to evaluate the anti-TNBC effect of LAF. Flow cytometry, wound healing, transwell assay, western blot, RT-PCR, and immunofluorescence analysis were carried out to investigate the pharmacological mechanism of LAF against TNBC.

**Results:**

LAF significantly inhibited the growth of MDA-MB-231 tumors, with downregulated tumor level of vimentin and upregulated level of ZO-1. In MDA-MB-231 and Hs-578T cells, LAF markedly suppressed cell proliferation, migration and invasion, and promoted apoptosis. Similarly, LAF increased the expression of ZO-1 and occludin proteins in MDA-MB-231 cells, and inhibited the expression of vimentin, snail and slug proteins in MDA-MB-231 and Hs-578T cells, as well as the expression of N-caderin in Hs-578T cells. Moreover, LAF also inhibited the phosphorylation of GSK-3β, thereby inhibited the downstream nuclear translocation of β-catenin and the expression of YAP. Furthermore, LAF significantly inhibited the expression of PI3K and AKT, and the phosphorylation of downstream mTOR.

**Conclusion:**

LAF showed anti-TNBC effect both *in vitro* and *in vivo.* Reversal of EMT by inhibiting GSK-3β/YAP/β-catenin signaling and PI3K/AKT/mTOR signaling contributes to the effect.

## Introduction

Breast cancer (BC), a malignant tumor arising in the epithelial tissue of the breast, is the tumor disease posing the greatest threat to women’s health today. According to the GLOBOCAN International Agency for Research on Cancer 2020, approximately 2.1 million women worldwide suffered from BC in 2020, accounting for about one-quarter of all tumor diseases in women and the highest number of tumor deaths in more than 100 countries (Bellanger et al., 2018; Sung et al., 2021). The incidence of BC is closely interrelated to the economic development of the country and the income level of the people, and according to relevant studies, some developing countries may become areas with a high incidence of BC (Bray et al., 2018). Therefore, the prevention and therapy of BC have become a top priority in the fight against oncological diseases in humans. Triple-negative breast cancer (TNBC) is one of the subtypes in BC, characterized by the lack of estrogen receptor, progesterone receptor, and human epithelial growth factor receptor 2 in breast tumor tissues. TNBC is a more aggressive subtype of BC, with the worst prognosis, heterogeneity, and highly aggressive (Bao and Prasad, 2019; Manjunath and Choudhary, 2021). In comparison to patients with non-triple-negative BC, patients with TNBC have worse survival (Li et al., 2017), which may be related to its high rate of metastasis (Tajbakhsh et al., 2019).

Anoikis was accidentally discovered in the early 1990s by an American scientist, Martin Schwartz, in an experiment (Frisch and Francis, 1994). It refers to a type of programmed cell death caused by the detachment of cells from the extracellular matrix (ECM). Anoikis is vital in organism development, disease development, and cancer metastasis. Cancer cell acquisition of anoikis resistance (AR) is the primary reason for tumor metastasis and invasion. For tumor metastasis, carcinomas can escape from the ECM and enter the lymphatic or blood circulation system, where they acquire AR through various molecular mechanisms to avoid apoptosis and colonize distant organs. In short, AR is the process by which carcinomas break away from the extracellular matrix, instead of undergoing apoptosis, enter the lymphatic or circulatory system and colonize distant organs, thus causing cancer metastasis (Dai et al., 2023). AR has two major characteristics, which are anchored non-dependent growth and epithelial-mesenchymal transition (EMT). Under normal conditions, EMT causes epithelial cells to remodel their cytoskeleton and separate from neighboring cells, thereby acquiring a dynamic phenotype. EMT plays an active role during wound healing, inflammation, or embryogenesis. However, in carcinomas, EMT occurs as one of the two main features of AR, and its occurrence represents the acquisition of tumor migratory and invasive properties (Kakavandi et al., 2018; Khan et al., 2022).

Traditional Chinese medicine (TCM) has a rich theoretical basis and clinical application for BC. Moreover, TCM may be defined as a long-term complementary and alternative therapy due to its unique advantages of multitargets and small side effects (Huemer et al., 2024; Yang et al., 2021; Yuan et al., 2022). *Fructus Arctii*, named Niubangzi in Chinese, is the dried mature fruit of *Arctium lappa* Linne; meanwhile, as a widely-used TCM which has the efficacy of detoxification, moistening the lung and throat abscess and removing carbuncle recorded in *YaoXingLun*, *XinxiuBencao, Bencaogangmu* and *ZhenZhuNang*, it is used for the treatment of cold, cough, measles, rubella, sore throat, erysipelas, carbuncle, etc. In China, people regard *A. lappa* L. as a healthy and nutritive food (Chan et al., 2011; Li et al., 2022; Yang 2022). Various chemical components such as lignans, volatile oils, fatty acids, terpenoids, and phenolic acids have been discovered in *F. arctii,* (Carlotto et al., 2016; Lee et al., 2011; Qin et al., 2014), and they have exhibited diverse pharmacological activities such as anti-tumor, anti-bacteria, anti-inflammation, immune modulation, and hypoglycemia (Annunziata et al., 2019; Gao et al., 2018; He et al., 2018; Li et al., 2022; Shabgah et al., 2021). Among them, Lappaol F (LAF) is a lignan that our group has discovered to inhibit the growth and promote apoptosis of various human carcinomas. The mechanisms behind this involve inducing cell cycle arrest in either the G1 or G2-M phase, up-regulating the expression of cell cycle proteins P21 and P27, and down-regulating the expression of Cyclin B1 and CDK1. Interestingly, LAF showed minimal toxicity to MCF10A human mammary epithelial cells at concentrations toxic to MCF-7 breast cancer cells (Sun et al., 2014). And in our previous study, we also found that LAF can induce S phase arrest in colorectal cancer cells by activating CDKN1C/p57, demonstrating its anti-tumor effects in the process (Yang et al., 2023) Meanwhile, in nude mice bearing human solid tumors formed by subcutaneous injection with cervical cancer cells (HeLa) or colon cancer cells (SW480), LAF significantly inhibited tumor growth. Further investigation revealed that LAF may work by inhibiting the protein expression and nuclear translocation of YAP (Li et al., 2021).

YAP and β-catenin are proteins whose activation promotes EMT, leading to cancer incidence and metastasis (Jiang et al., 2020). Studies have proven that nuclear YAP can upregulate β-catenin expression, altering the downstream effectors of both the Hippo and Wnt pathway. These alterations activate the transcription of EMT-TFs, which regulate the expression of EMT markers, activate metastasis-related proteins and anti-apoptotic proteins, suppress pro-apoptotic proteins, and enable cancer cells to acquire AR (Gonzalez and Medici, 2014; Imajo et al., 2012). In addition, YAP can mediate the interaction between the Hippo and PI3K/AKT pathway (Qian et al., 2021). PI3K/AKT is one of the most frequently altered signaling pathways in human cancers, supporting the activation of numerous proteins sustaining cell proliferation and aggressiveness. Some studies claimed that the PI3K/AKT pathway can regulate the YAP/TAZ (Simula et al., 2022).

In this study, we aimed to investigate if LAF exerts an anti-TNBC effect *in vivo* and to explore the potential regulation effect on YAP/β-catenin and PI3K/AKT pathway by focusing on EMT.

## Materials and methods

### Chemicals and reagents

**FIGURE 1:**
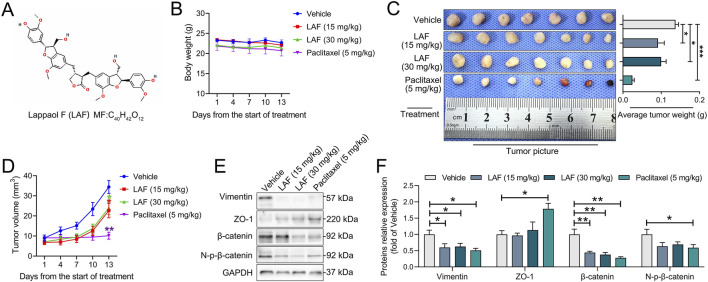
LAF inhibited the growth and promoted the MET of MDA-MB-231-xenografts·in·NCG·mice, the mice were divided into the vehicle, LAF-administration group (15 mg/kg and 30 mg/kg) and positive control group (paclitaxel, 5 mg/kg). **(A)** structure of LAF. **(B)** body weights (n = 7). **(C)** tumor pictures and tumor weight (n = 7). **(D)** tumor volumes (n = 7). **(E)** representative western blots showing the expression of EMT-related proteins changes and **(F)** normalized to GAPDH (n = 3). Data were expressed as mean ± SEM. Compared to the vehicle group, data were significantly different at ^*^p < 0.05, ^**^p < 0.01, and ^***^p < 0.001.

LAF (Figure 1A) was obtained from the seeds of *F. arctii* as previously described (Sun et al., 2014). Paclitaxel Injection was the product of Hospira Australia Pty Ltd. Verteporfin (VP) was purchased from Selleck Chemical (Houston, United States). The dissolution methods of LAF and VP were consistent with those previously described (Li et al., 2021). Cell Counting Kit-8 (CCK-8) was obtained from Fude Biological Technology Company (Hangzhou, China), and the annexin V-FITC/PI apoptosis kit was purchased from Beyotime (Shanghai, China). Crystalline violet was obtained from Macklin (Shanghai, China). Matrigel was purchased from Corning (NY, United States). The antibodies and primer used in this study are shown in Tables 1, 2.

**TABLE 1:** Antibodies used in Western blot analysis.

Antibody	Manufacturer	Catalog no.	Dilution
Anti-Vimentin	CST	5741	1:3,000
Anti-ZO-1	CST	13663	1:1,000
Anti-N-cadherin	CST	13116	1:3,000
Anti-Vimentin	CST	5741	1:3,000
Anti-GSK-3β	CST	12456	1:3,000
Anti-p-GSK-3β(Ser9)	CST	5558S	1:1,000
Anti-β-catenin	CST	8480	1:3,000
Anti-non-phosphor (Active) β-Catenin (Ser33/37/Thr41)	CST	8814	1:1,000
Anti-YAP	CST	14074	1:3,000
Anti-PI3K	CST	4228	1:3,000
Anti-AKT	CST	4685	1:3,000
Anti-p-mTOR (Ser2448)	CST	5536	1:1,000
Anti-PTEN	CST	9188	1:3,000
Anti-p-AKT (Ser473)	CST	4060	1:1,000
Anti-GAPDH	CST	5714	1:3,000
Anti-Snail and Slug	Abcam	Ab180714	1:1,000
Anti-Occludin	Abcam	Ab21632	1:1,000
Anti-rabbit secondary antibody	Abcam	Ab6721	1:20,000

**TABLE 2:** Primers used in the RT-PCR analysis.

Gene	Forward primer sequence (5′-3′)	Reverse primer sequence (5′-3′)
YAP	TAG​CCC​TGC​GTA​GCC​AGT​TA	TCA​TGC​TTA​GTC​CAC​TGT​CTG​T
β-catenin	AGC​GAG​CAG​CCC​CCA​AAG​TT	GGG​CAC​GAA​GGC​TCA​TCA​TT
Slug	CGA​ACT​GGA​CAC​ACA​TAC​AGT​G	CTG​AGG​ATC​TCT​GGT​TGT​GGT
Vimentin	GAC​GCC​ATC​AAC​ACC​GAG​TT	CTT​TGT​CGT​TGG​TTA​GCT​GGT
LEF1	AGA​ACA​CCC​CGA​TGA​CGG​A	GGC​ATC​ATT​ATG​TAC​CCG​GAA​T
GSK-3β	GGC​AGC​ATG​AAA​GTT​AGC​AGA	GGC​GAC​CAG​TTC​TCC​TGA​ATC
GAPDH	AGGTGAAGGTCGGA	TTGAGGTCAATGAAG

### Cell lines and cell culture

Human triple-negative breast cancer lines MDA-MB-231 and Hs-578T were obtained from The Cell Bank of the Chinese Academy of Sciences, Shanghai, China. Both cell lines were cultured in Dulbecco’s Modified Eagle Medium (DMEM) supplemented with 10% fetal bovine serum (FBS), at 37°C under a humidified atmosphere with 5% CO_2_. Reagents used for cell culture were purchased from Gibco Life Technologies (Grand Island, United States)

### *In vivo* study

35-day-old NCG (NOD-Prkdcem26Cd52Il2rgem26Cd22/Nju) mice were purchased from Nanjing Biomedical Research Institute of Nanjing University. Animal feeding and the experimental operation were conducted in the Animal Experimental Center of the First Affiliated Hospital of Sun Yat-Sen University [License Number: SYXK (Yue) 2015-0108] with the environmental conditions of specific pathogen-free, 22°C ± 1°C, 50%–65% of humidity and 12h/12 h of light/dark rhythm. The experiment was approved by the ethics committee of the University, and the document number is [2018]248.

To assass the *in vivo* anti-TNBC effect of LAF, MDA-MB-231 cells suspension at a density of 7 × 10^7^ cells/mL in PBS were inoculated into the fourth right breast pad of the mice (50 μL per mouse) to establish a breast cancer *in situ* tumor model. Twenty days after inoculation, mice with good tumor growth and moderate tumor size (3–4 mm in diameter) were randomly divided into 4 groups (N = 7 in each group). They were then intravenously given 15 mg/kg or 30 mg/kg of LAF, 5 mg/kg of Paclitaxel Injection (positive control), or equivalent volume of the vehicle. LAF and vehicle were administered daily for 12 consecutive days, while Paclitaxel Injection was given every other day for 5 times. Body weight and tumor size were measured on days 0, 3, 6, 9, and 12. After the treatment, mice were euthanized with ether, and tumors were collected for analysis.

### CCK-8 assay for cell proliferation ability

TNBC cells were seeded in 96-well plates at a density of 8 × 10^3^ cells/well, incubated with various LAF concentrations for 24 h, 48 h, or 72 h. After incubation, the cells were subjected to a CCK-8 assay following the manufacturer’s instructions, and the absorbance in each well was measured at 450 nm using a microplate reader. Wells containing untreated cells served as the control, while wells containing only medium without cells were considered as blank. The proliferation inhibition rate (%) was calculated using the following equation:

$$\text{Inhibition rate }\left(\%\right)=\left({\text{OD}}_{\text{Control}}-{\text{OD}}_{\text{Test}}\right)\;/\;\left({\text{OD}}_{\text{Control}}-{\text{OD}}_{\text{Blank}}\right)\times 100\%$$

### Apoptosis assay

We used an Annexin V-FITC/PI Apoptosis Kit to assess the apoptosis rate of TNBC cells when exposed to LAF. Initially, MDA-MB-231 and Hs-578T cells were plated in 3.5 cm culture dishes at a density of 1 × 10^5^ cells per dish and left to incubate overnight. Subsequently, the cells were treated with LAF at varying concentrations (10, 25, or 50 μM) for different durations (24 h, 48 h, or 72 h). Following treatment, the cells were harvested and processed according to the manufacturer’s instructions of the kit. Flow cytometry analysis was carried out using a Beckman Coulter Commercial Enterprise flow cytometer in LA, United States, and the percentage of apoptotic cells was determined using Flowjo10.6.2 software.

### Wound healing assay

The TNBC cells were plated in 6-well plates at a density of 1 × 10^5^ cells/well) with or without LAF treatment (10, 25, or 50 μM) for 24 h. Before treatment, scratches were made onto the plates using 200 μL pipette tips. After the cells adhered, six random fields of view were selected and photographed at 0 and 24 h using an inverted microscope (10×). The area of wounds was then analyzed using ImageJ software, and the percentage healing rates were calculated using the following formula:

$$\text{Wound healing assay }\left(\%\right)=\frac{\text{scratch}\;\text{area}\;\left(0\;h\right)‐\;\text{scratch}\;\text{area}\;\left(24\;h\right)}{\text{scratch}\;\text{area}\;\left(0\;h\right)}\times 100\%$$

### Transwell assay

MDA-MB-231 and Hs-578T cells were prepared at a density of 2.5 × 10^5^ cells/mL and suspended in serum-free medium with or without LAF (10, 25, or 50 μM). These cells were then seeded into the upper chamber of an 8-micron transwell. The transwell’s filters were coated with a layer of diluted matrigel, and the lower chambers were filled with a medium containing 15% FBS, maintaining an equivalent concentration of LAF as the upper chamber. Following an incubation period of 24 h, any cells remaining on the upper surface of the chamber were carefully removed. The cells that had migrated to the lower surface were fixed using 4% paraformaldehyde for a half-hour and subsequently stained with 0.1% crystal violet for 20 min. The invaded cells were then visualized and captured using a light microscope (20×), and the cell count was determined using ImageJ software. The cell invasion rate was subsequently computed using the following formula:

$$\text{Invasion radio }\left(\%\right)=\frac{cells\;amount\left(LAF\right)}{cells\;amount\left(control\right)}\times 100\%$$

### Western blot analysis

Total protein samples were extracted from tumors or cultured cells after indicated treatments. Protein concentrations were determined using the BCA protein assay kit. SDS-polyacrylamide gels were then prepared, and equal amounts of protein samples were loaded onto polyvinylidene fluoride (PVDF) membranes. These membranes were blocked with 5% skimmed milk or 5% bovine serum albumin (BSA) in TBST at room temperature for 1 h. Following blocking, the membranes were incubated with primary monoclonal antibodies at 4°C overnight. The PVDF membranes were then washed 3 times for 10 min each in TBST and incubated with HRP-conjugated secondary antibodies at room temperature for 1 h. Protein bands were detected using an enhanced ECL detection method. The antibodies used are listed in Table 1.

### Immunofluorescence analysis

MDA-MB-231 cells (2 × 10^4^ cells/well) were seeded into 24-well plates containing preplaced cell slides and incubated at 37°C for 24 h under a humidified atmosphere with 5% CO_2_. Then, the original medium in each well was discarded and replaced with fresh medium infused with 50 μM LAF, 100 μM VP, or a combination of both. The plates were then incubated for an additional 48 h. Subsequently, the slides were washed with PBS for 5 min 3 times. Cells on the slides were fixed with 4% paraformaldehyde for 15 min and washed with PBS thoroughly as previously described. Then, cells were permeabilized using 0.2% Triton-100 at room temperature for 20 min, blocked with 1% BSA for 30 min, and exposed to the corresponding antibody of β-catenin (dilution 1:100) in 1% BSA at 4°C overnight, followed by the previously mentioned washing procedure. A fluorescence-conjugated secondary antibody was then applied to track the primary antibody for 1 h at room temperature. Finally, in a dark setting, a DAPI-containing mounting medium was added to the slides, and images were captured using a confocal microscope.

### Reverse transcription–quantitative polymerase chain reaction (RT-PCR) analysis

MDA-MB-231 cells were subjected to the indicated treatment, before which total RNA samples were extracted. In general, the extraction process involved washing the cells twice with PBS, collecting them into a 1.5 mL EP tube, and then extracting with Trizol (Life Technologies, MD, United States). The RNA concentration was then measured using a spectrophotometer (Thermo Fisher Scientific, Shanghai, China). Subsequently, the RNA was reverse transcribed into cDNA using a reverse transcription kit (Takara Biomedical Technology Co.Ltd., Dalian, China). Finally, the mRNA expression level was determined using a PCR kit (Takara Biomedical Technology Co.Ltd., Dalian, China) in a fluorescence ratio PCR instrument (Bio-rad, LA, United States), following the manufacturer’s instructions. The specific primer sequences used are listed in Table 2.

### Statistical analysis

Statistical analysis was performed using IBM SPSS Statistics software, version 26.0 (SPSS, Inc., Chicago, IL, United States). All data are represented as the mean ± SEM, and each experiment was repeated at least three times to ensure consistency. Intergroup differences were determined using one-way ANOVA, followed by Tukey’s test for post-hoc analysis. A *p ≤ 0.05* was considered statistically significant.

## Results

### LAF inhibited the growth of MDA-MB-231 xenografts in NCG mice

Anti-TNBC effects of LAF and Paclitaxel Injection on MDA-MB-231 tumor-bearing NCG mice are presented in Figures 1B–D. As anticipated, treatment with 5 mg/kg paclitaxel nearly eradicated tumor growth. While treatment with 15 mg/kg/d or 30 mg/kg/d of LAF failed to stop tumor growth but did decelerate its progression, exhibiting an *in vivo* anti-TNBC effect without impacting the body weight of mice. However, a dose-dependent anti-tumor effect of LAF was not observed. Expression of EMT-related proteins was investigated using Western blotting. As shown in Figures 1E, F, tumors in the vehicle group expressed a high level of vimentin (a mesenchymal marker) but a very low level of ZO-1 (an epithelial marker), showing EMT characteristics. Treatment with LAF or paclitaxel significantly downregulated the expression level of vimentin but upregulated the expression level of ZO-1, indicating that the treatments reversed EMT, thereby promoting the mesenchymal-epithelial transition (MET) in tumor cells.

### LAF inhibited proliferation and promoted apoptosis in TNBC cells

**FIGURE 2:**
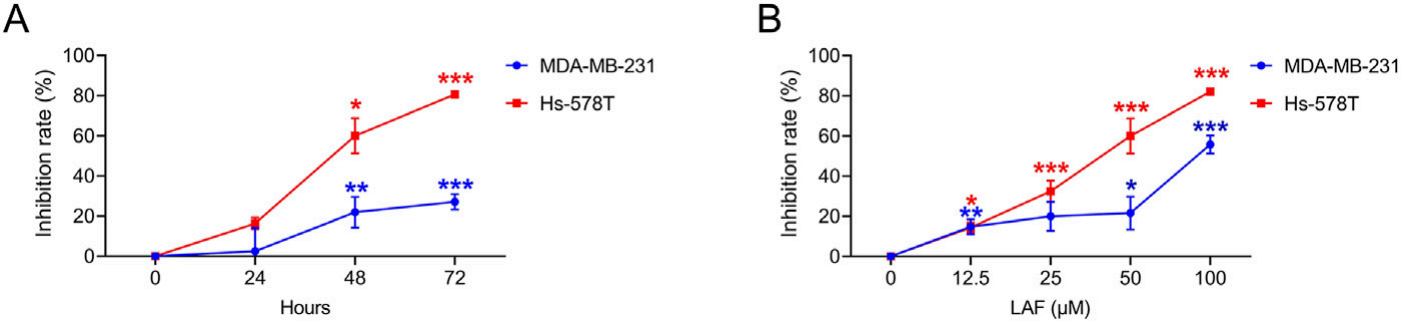
LAF inhibited the proliferation of TNBC cells. Cells were treated with 50 μM LAF for 24, 48, or 72 h **(A)**, or with different concentrations of LAF for 48 h **(B)**. The OD was measured by CCK-8 assay. Proliferation inhibition rate (%) = (OD_Control_ - OD_Test_)/(OD_Control_ - OD_Blank_) × 100%. Data are expressed as the mean ± SEM (n = 3). Compared to Control, data were significantly different at ^*^p < 0.05, ^**^p < 0.01, and ^***^p < 0.001.

**FIGURE 3:**
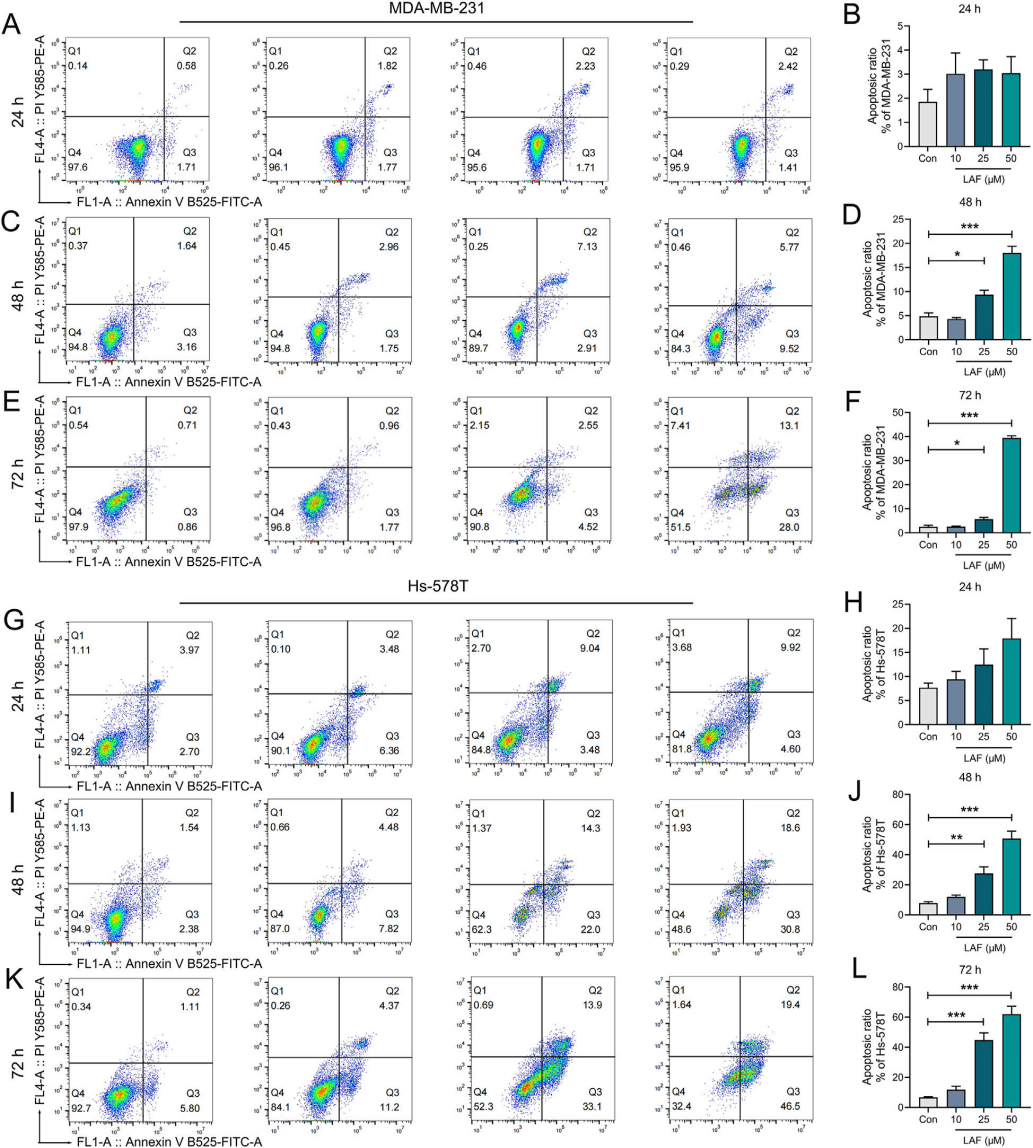
Time- and dose-dependent effects of LAF on the apoptosis of TNBC cells. MDA-MB-231 cells **(A–F)** and Hs-578T cells **(G–L)** after the indicated treatment were stained with Annexin V-FITC/PI and then analyzed by flow cytometry and the rates of apoptotic cells (including early apoptotic and late apoptotic cells) were calculated using Flowjo10.6.2 software. All data are expressed as the mean ± SEM (n = 3). Compared to Control (Con), data were significantly different at ^*^p < 0.05, ^**^p < 0.01, and ^***^p < 0.001.

Showing the anti-TNBC effect and the ability to reverse EMT *in vivo*, LAF was then investigated to ensure it works on tumor cells directly. The direct anti-TNBC effect of LAF was evaluated in cultured MDA-MB-231 and Hs-578T cells, with high EMT characteristics (Blick et al., 2008). As shown in Figure 2, LAF exhibited a time- and dose-dependent inhibitory effect on tumor cell growth but was relatively less effective in MDA-MB-231 cells, with the IC_50_ values (48 h) of 59.32 ± 1.94 μM (MDA-MB-231) and 35.33 ± 2.06 μM (Hs-578T), respectively. Effects of LAF on the apoptosis of MDA-MB-231 and Hs-578T cells were also investigated. As expected, LAF showed time- and dose-dependent ability to induce apoptosis in both cells, it was more effective in Hs-578T cells (Figure 3).

### LAF inhibited the migration and invasion of TNBC cells by inhibition of EMT features

**FIGURE 4:**
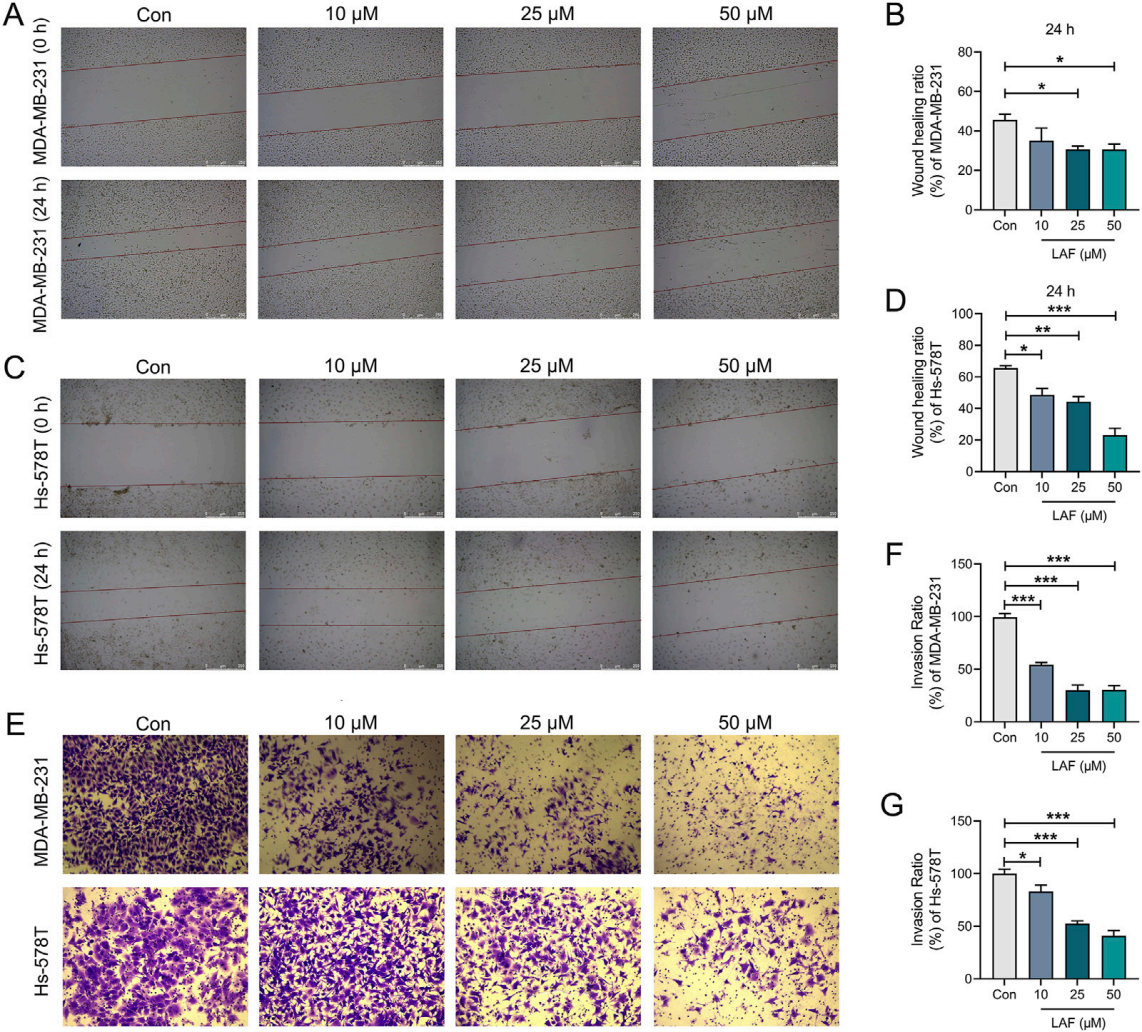
Effects of LAF on the migration and invasion of TNBC cells (24 h). **(A, B)** Wound healing assay and ratio of MDA-MB-231 cells, scale bar: 250 μm. **(C, D)** Wound healing assay and ratio of Hs-578T cells, scale bar: 250 μm. **(E)** Transwell assay of MDA-MB-231 and Hs-578T cells, MDA-MB-231 and Hs-578T cells in the lower chamber were stained with crystal violet, scale bar: 75 μm. **(F, G)** Invasion ratio of MDA-MB-231 and Hs-578T cells. Data are expressed as the mean ± SEM (n = 3). Compared to Control group, data were significantly different at ^*^p < 0.05, ^**^p < 0.01, and ^***^p < 0.001.

Migration and invasion are hallmarks of malignant tumors, with EMT serving as a crucial early stage in the malignant transformation of tumor cells. This process facilitates the detachment of tumor cells from the primary tissue and their entry into the circulatory system. Drugs that target the EMT process could potentially inhibit the systematic metastasis of carcinomas, thereby prolonging patient survival. Effects of LAF on the migration and invasion capabilities of TNBC cells were assessed via wound healing assay and Transwell assay after 24 h, respectively. The wound healing assay (Figures 4A–D) demonstrated that incubation with 25 or 50 μM LAF for 24 h significantly inhibited wound healing in both cell lines. Concurrently, the Transwell assay (Figures 4E–G) indicated that the addition of 10, 25, or 50 μM LAF markedly reduced the number of tumor cells in the lower chamber. The results suggested that LAF directly prevents the migration and invasion of MDA-MB-231 and Hs-578T cells.

**FIGURE 5:**
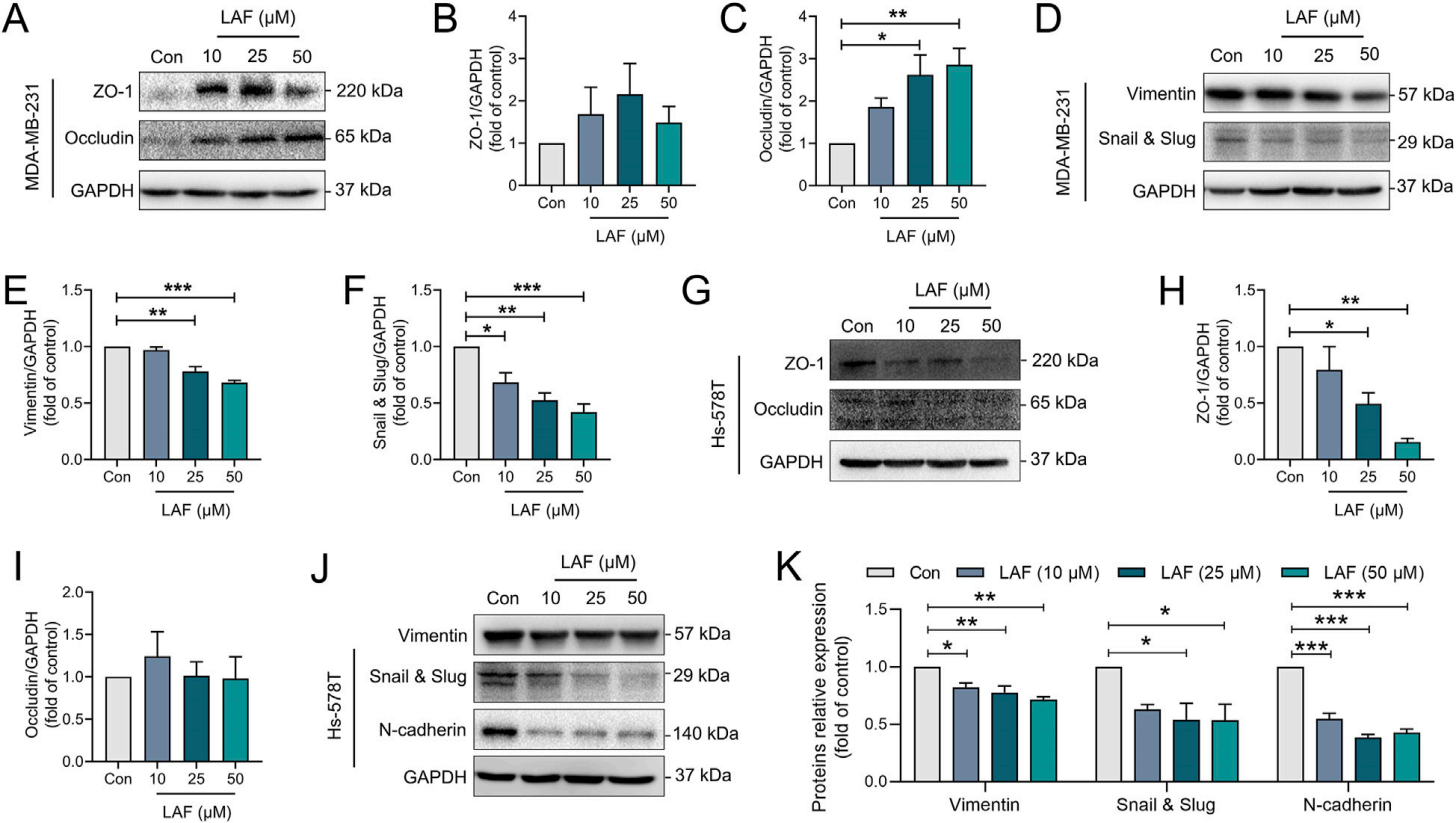
LAF regulated EMT-associated proteins in TNBC cells. **(A–F)** Representative western blots showing the expression of EMT-related proteins changes and normalized to GAPDH in MDA-MB-231 cells (n = 3). **(G–K)** Representative western blots showing the expression of EMT-related proteins changes and normalized to GAPDH in Hs-578T cells (n = 3). Data were expressed as mean ± SEM. Compared to Con, data were significantly different at ^*^p < 0.05, ^**^p < 0.01, and ^***^p < 0.001.

Further investigation was conducted to examine the influence of LAF on the expression of EMT-associated proteins, with the results depicted in Figure 5. As expected, MDA-MB-231 cells expressed very high levels of mesenchymal marker protein (vimentin) but very low levels of epithelial markers (ZO-1 and occludin). Similarly, Hs-578T cells expressed high levels of mesenchymal markers (vimentin and N-cadherin) and EMT transcription factor Snail and Slug (EMT transcription factor), indicating mesenchymal features and high malignancy. After treatment with LAF, the expression levels of vimentin and Snail and Slug in both cell lines were downregulated. Additionally, N-cadherin expression in Hs-578T cells was downregulated. Occludin and ZO-1 levels in MDA-MB-231 cells were also modulated. These outcomes suggest that LAF targeted the EMT process, promoting mesenchymal-epithelial transition in both cell lines. However, the mechanism behind the LAF-induced downregulation of ZO-1 expression in Hs-578t cells remains to be elucidated.

### LAF inhibited the YAP/β-catenin signaling in TNBC cells

**FIGURE 6:**
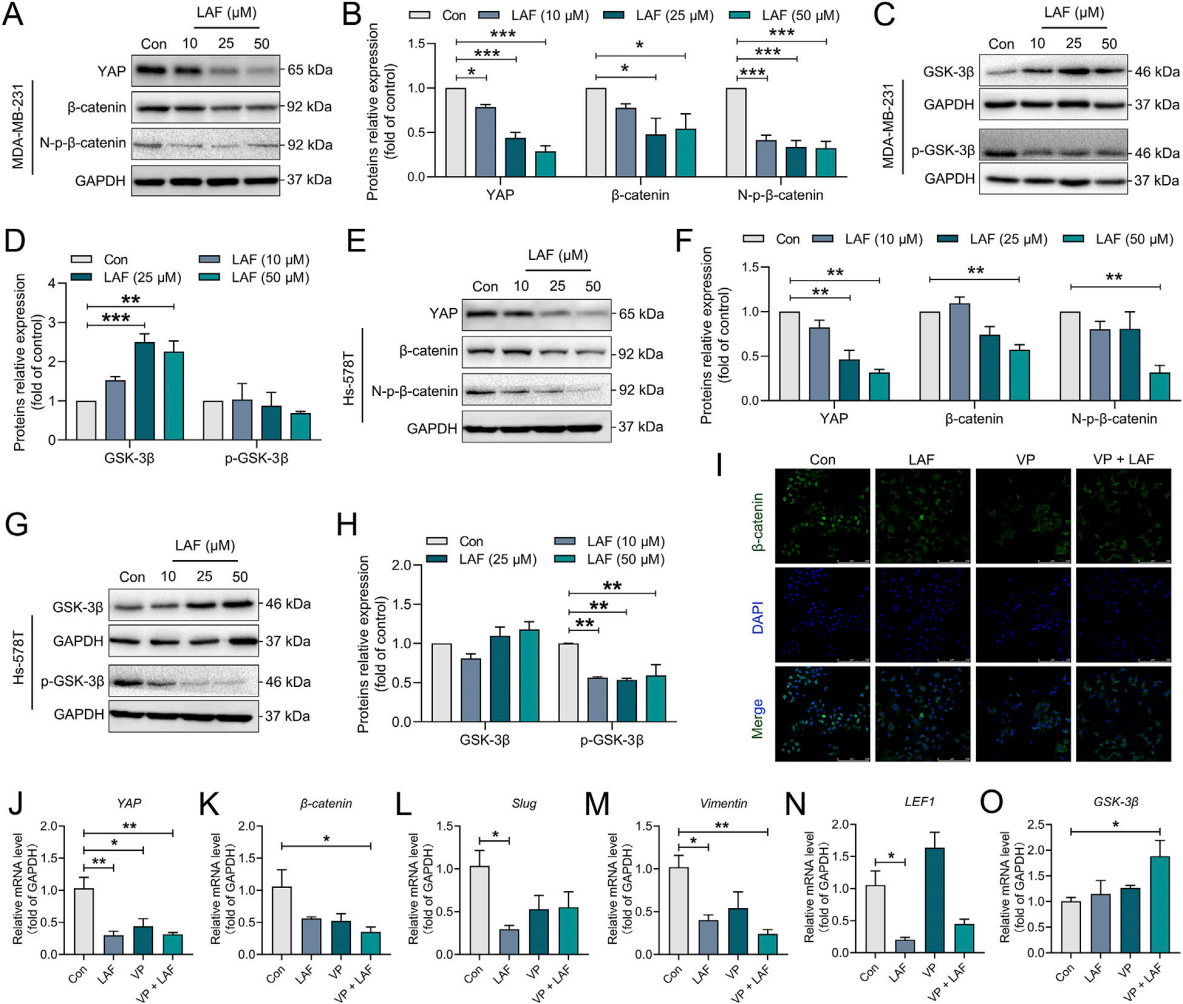
LAF inhibited the YAP/β-catenin signaling in TNBC cells. **(A–D)** Representative western blots showing the expression of YAP/β-catenin signaling proteins changes and normalized to GAPDH in MDA-MB-231 cells. **(E–H)** Representative western blots showing the expression of YAP/β-catenin signaling proteins changes and normalized to GAPDH in Hs-578T cells. **(I)** Subcellular localization of β-catenin in MDA-MB-231 cells was detected by immunofluorescent staining. **(J–O)** RT-PCR detected the mRNA expression levels of the YAP/β-catenin signaling and its downstream EMT-related genes in MDA-MB-231. Data are expressed as the mean ± SEM (n = 3). Compared to Con, data were significantly different at ^*^p < 0.05, ^**^p < 0.01, and ^***^p < 0.001.

YAP functions as a transcription co-activator by binding to the transcriptional factors in the Hippo signaling pathway in the nucleus, and as a regulator by binding to another transcriptional co-activator, β-catenin, of the Wnt signaling pathway (Jiang et al., 2020). In this study, the effects of LAF on the YAP/β-catenin pathway in TNBC cells were examined. As shown in Figures 6A–H, MDA-MB-231 and Hs-578T cells expressed high levels of YAP, total β-catenin and non-phosphorylated β-catenin (N-p-β-catenin, the activated form). These cells also had low levels of GSK-3β (which inactivates β-catenin via phosphorylation) and high levels of phosphorylated GSK-3β (p-GSK-3β, the inactivated form), suggesting inhibited GSK-3β activity and a normally functioning YAP/β-catenin pathway. With LAF treatment, cellular levels of YAP, β-catenin, N-p-β-catenin and p-GSK-3β were reduced, while the GSK-3β levels increased in dose-dependent manner. The indicates that LAF can weaken EMT in TNBC cells by inhibiting the YAP/β-catenin pathway through the activation of GSK-3β. Immunofluorescent staining of the β-catenin in MDA-MB-231 cells (Figure 6I), the reduced fluorescence intensity in the cytoplasm and nucleus by VP (a known YAP inhibitor) was observed, indicating that there is a causal relationship between YAP activation and β-catenin activation; LAF inhibited β-catenin expression in a manner similar to VP. These results support that LAF inhibited both the protein expression and nuclear translocation of β-catenin in TNBC cells by inhibiting YAP. Consistent with the above results, in LAF-treated MDA-MB-231 cells, downregulated mRNA levels of EMT genes including *YAP*, *β-catenin*, *Slug, Vimentin*, and transcription factor gene *LEF1* were observed, as well as upregulated *GSK-3*β genes (Figures 6J–O). In addition, when the activity of GSK-3β was inhibited by IM-12 (Selleck, Shanghai, China), the inhibitory effect of 50 μM LAF on Vimentin was more significant (Supplementary Figure SA), suggesting that LAF may affect the EMT process by regulating the activity of GSK-3β. Besides, the proliferation of MDA-MB-231 was inhibited by the application of these inhibitors, and the inhibition was more pronounced in combination with LAF (Supplementary Figure SB). In brief, these results provide evidence that the LAF weakened the EMT and growth of TNBC cells via inhibition of the YAP/β-catenin pathway through activation of GSK3β. Similar results were observed in the animal experiments described above (Figures 1D, E).

### LAF regulated PI3K/AKT signaling in TNBC cells

**FIGURE 7:**
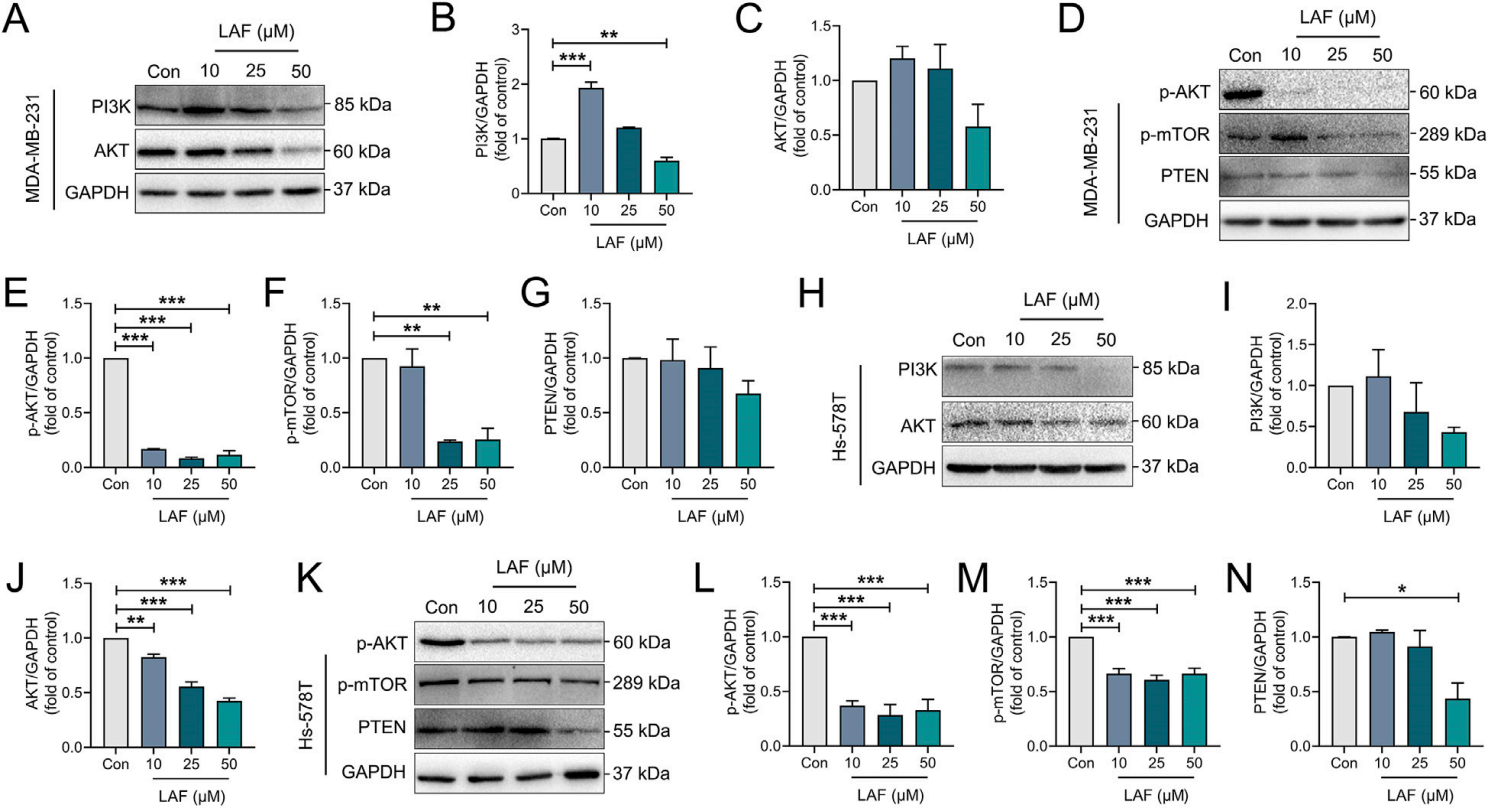
LAF showed regulatory effects on the PI3K/AKT pathway in TNBC cells. **(A–G)** Representative western blots showing the expression of PI3K/AKT signaling proteins changes and normalized to GAPDH in MDA-MB-231 cells. **(H–N)** Representative western blots showing the expression of PI3K/AKT signaling proteins changes and normalized to GAPDH in Hs-578T cells. Data are expressed as the mean ± SEM (n = 3). Compared to Con, data were significantly different at ^*^p < 0.05, ^**^p < 0.01, and ^***^p < 0.001.

The PI3K/AKT pathway, an upstream regulator of YAP/β-catenin, inhibits GSK-3β activity via phosphorylation and is a known triggering mechanisms of AR by promoting EMT (Glaviano et al., 2023). In this study, the effects of LAF on the PI3K/AKT pathway in TNBC cells were also investigated. As shown in Figure 7, treatment with LAF (10, 25, or 50 μM) for 48 h significantly reduced both the expression and phosphorylation of AKT in both MDA-MB-231 and Hs-578T cells. Consequently, decreased phosphorylation of the downstream serine/threonine-protein kinase mTOR was observed in both cell lines. Despite these changes, enhanced expression of PTEN which is a negative regulator of PI3K/AKT signaling was not observed, suggesting that PTEN is not involved in the inhibition of PI3K/AKT signaling by LAF. And a PI3K and AKT inhibitor, Miltefosine (Selleck, Shanghai, China) was applied to MDA-MB-231 for 48 h to further confirmation of whether LAF regulates the EMT process via PI3K/AKT. As the results are shown in the Supplementary Figure, when the PI3K or AKT activities were inhibited, the suppression of vimentin and N-cad by LAF was more pronounced, compare with the absence of LAF. These results above suggested that PI3K/AKT/mTOR signaling may play a role in the regulation of YAP and β-catenin via GSK3β.

## Discussion

In this study, we have identified a plant-derived antitumor agent LAF and uncovered its TNBC inhibitory potential using TNBC cell lines and related animal model. Our results indicate that in MDA-MB-231 xenografts tumor, LAF exerts a strong growth inhibition. In addition, animals seemed to tolerate the treatment of LAF without significant body weight changes during treatment period, suggests that LAF may not have toxic effects on the body at this dose (15 mg/kg and 30 mg/kg). However, we only carried out observation on whether LAF has any effect on the organism superficially, but we did not test the detailed toxic side effects of LAF, such as biochemical indexes, etc. Therefore, in the future, we will further set up relevant toxicity experiments to explore the possible potential adverse effects of LAF. Othersides, our studies indicate that LAF shown a inhibitory effect of proliferation, migration and invasion, and also triggers apoptosis in TNBC cell lines. In which, LAF exerts a significantly stronger anticancer effect in Hs-578T cells compared to MDA-MB-231 cells. It will guide us to in-depth studies on Hs-578T cells in the future to provide a more scientific basis for the treatment of TNBC with LAF. Thurs, our results, for the first time, indicate that LAF has a tumor suppression function *in vitro* and *in vivo*, which suggest LAF has a great potential to be developed as an anti-TNBC therapeutic.

The acquisition of AR by tumor cells is the prerequisite for tumor metastasis and invasion with EMT being one of the key features of AR. During EMT, cancer cells activate epigenetic pathways, downregulate cell-cell adhesion molecules such as E-cadherin and γ-catenin, and upregulate mesenchymal markers likes vimentin, fibronectin, α-smooth muscle actin (SMA), N-cadherin, and MMPs. These changes lead to phenotypic transformations in the cells, enabling epithelial cells to acquire a mesenchymal-like state, thereby detaching from the primary tumor site and migrate to invade distant tissues. Consequently, the acquisition of a mesenchymal phenotype is thought to be positively correlated with the ability of carcinoma cells to overcome anoikis (Liu et al., 2023; Simpson et al., 2008). Besides this, a series of EMT-TFs are also involved in regulating the EMT process, including ZEB1/2, Snail/Slug, Twist, etc. (Goossens et al., 2017). They are often aberrantly expressed in tumors, causing downregulated expression of E-cadherin and upregulated expression of mesenchymal markers (Das and Law, 2018). In our study, MDA-MB-231 and Hs-578T TNBC cells exhibited high levels of mesenchymal markers and low levels of epithelial markers, displaying distinct EMT features, such as spun-conical shape. This is in contrast to cancer cells with less distinct EMT features, such as gastric cancer cells and colon cancer cells (Sheng et al., 2020; Song et al., 2021; Yuan et al., 2019; Zhang et al., 2020). Additionally, pre-experimental results confirmed AR: cells inoculated in 96-well plates coated with poly-HEME gel (so that the cells could not grow against the wall) grew and proliferated normally in a detection period of 30 days (data not shown). Cells with pronounced EMT characteristics exhibit high malignancy and poor treatment prognosis. Pharmacological interventions that inhibit tumor mesenchymal marker expression and upregulate epithelial marker expression can induce MET, restoring epithelial characteristics and reducing malignancy, thereby enhancing the therapeutic effect. In our study, LAF significantly inhibited the mesenchymal signature protein levels and promoted the epithelial signature protein levels in MDA-MB-231 xenografts tumor, showing LAF can reverse EMT *in vivo.* In addition, LAF significantly downregulated mesenchymal signature protein levels in MDA-MB-231 and Hs-578T cells and upregulated epithelial signature protein levels in MDA-MB-231 cells. These results suggesting that the inhibitory effect of LAF on the migration and invasion of TNBC should be achieved by inducing the MET.

YAP is the main effector molecule of the Hippo signaling pathway and a downstream effector molecule of the atypical Wnt pathway. Abnormal expression of YAP and β-catenin often leads to the cancer occurrence and metastasis. GSK-3, an upstream effector molecule of β-catenin, may affect YAP function and plays a regulatory role in various cellular processes, including glycogen synthesis,etc. GSK-3 activity is regulated by the phosphorylation of serines (Ser21/Ser9/a/b isoforms) at conserved sequences near the amino terminus of the protein. GSK-3 primarily phosphorylates Ser33 and Ser37 of β-catenin, disrupting complexes formed by β-catenin and other proteins such as YAP. Moreover, the phosphorylation of specific sites on β-catenin is related to the EMT process; for example, phosphorylation at Y654 prevents β-catenin from binding to E-cadherin, while phosphorylation of Y489 inhibits its binding to N-cadherin (Song et al., 2021).

In turn, YAP activity is affected by the cellular environment, such as cell junction formation and cell polarity (Sheng et al., 2017). Tight junction and adherent junction degradation induce YAP entry into the nucleus to target gene expression (Sheng et al., 2020). Tight junction and adherent junction are closely related to the EMT process. Several components of tight junctions are involved in regulating Hippo and YAP activity (Zhang et al., 2020). For example, ZO-2 induces YAP entry into the nucleus, while ZO-1 inhibits the activity of TAZ, a homolog of YAP (Guha et al., 2019). In both normal and cancer stem cells, the activation of WNT signaling and involvement of β-catenin lead to changes in the expression of EMT-associated cell adhesion molecules. β-catenin links E-cadherin to the actin cytoskeleton, forming an E-cadherin/β-catenin complex (Tafrihi and Nakhaei Sistani, 2017). Therefore, regulating YAP/β-catenin to induce MET in malignant cells by regulating would help control further cancer progression.

The effect of LAF on the mRNA expression of *YAP*, β*-catenin*, and *GSK3*β in MDA-MB-231 cells mirrored its impact on the corresponding protein expression. Additionally, its effect on the mRNA expression of EMT-related genes and transcription factors downstream of the YAP/β-catenin pathway was consistent with its effect on the expression of the related proteins. This further supports the evidence that LAF can inhibit EMT in MDA-MB-231 cells by regulating the YAP/β-catenin pathway.

The PI3K/AKT pathway, a crucial signaling pathway promoting cell survival, integrates signals from integrin and growth factor receptors and is a common mechanism underlying of AR (Peng et al., 2022; Xiao et al., 2021). For example, AKT activation modulates the activity of certain transcription factors, thus influencing the expression of pro-apoptosis proteins and anti-apoptosis proteins. Additionally, persistent AKT activation follows the upregulation of N-cadherin expression, signifying the occurrence of EMT in cancer epithelial cells. It has also been demonstrated that N-cadherin facilitates PI3K recruitment, which subsequently activates AKT and induces AR (Xu et al., 2015). In our study, LAF showed the ability to inhibit the PI3K/AKT/mTOR pathway in TNBC cells. However, how the specific mechanism through which LAF acts on this pathway, and whether it affects AR via this pathway, remain unclear and warrant further investigation.

## Conclusion

The results of this study suggest that LAF has a potential on anti-TNBC *in vivo* and *in vitro*. And the underlying mechanisms involved the inhibition of the GSK-3β/YAP/β-catenin and PI3K/AKT pathways, which weakened the mesenchymal features but enhanced the epithelial features. This study contributes to elucidate the pharmacological mechanism of the anti-TNBC activity of LAF and reveal the potential mechanism of LAF on TNBC.

## Data Availability

The original contributions presented in the study are included in the article/Supplementary Material, further inquiries can be directed to the corresponding authors.

## Abbreviations

AIG, Anchorage-independent growth; AKT, receptor serine/threonine kinases; AR, Anoikis resistance; BC, Breast Cancer; BL1, Basal-like 1; BL2, Basal-like 2; ECM, Extracellular Matrix; EMT, Epithelial-mesenchymal transition; EMT-TFs, EMT transcription factors; GSK-3β, glycogen synthase kinase 3 beta; LAF, Lappaol F; MET, Mesenchymal-epithelial transition; PI3K, phosphatidylinositol 3-kinase; TNBC, Triple-negative breast cancer; VP, Verteporfin; YAP, Yes-associate protein.
