# Supplementary figures and images for: Anti-TNBC effects of Lappaol F by targeting epithelial-mesenchymal transition via regulation of GSK-3β/YAP/β-catenin and PI3K/AKT pathways
Source: Front Pharmacol. 2025 Feb 7;16:1496511. doi: 10.3389/fphar.2025.1496511 (PMC11842333; doi:10.3389/fphar.2025.1496511)

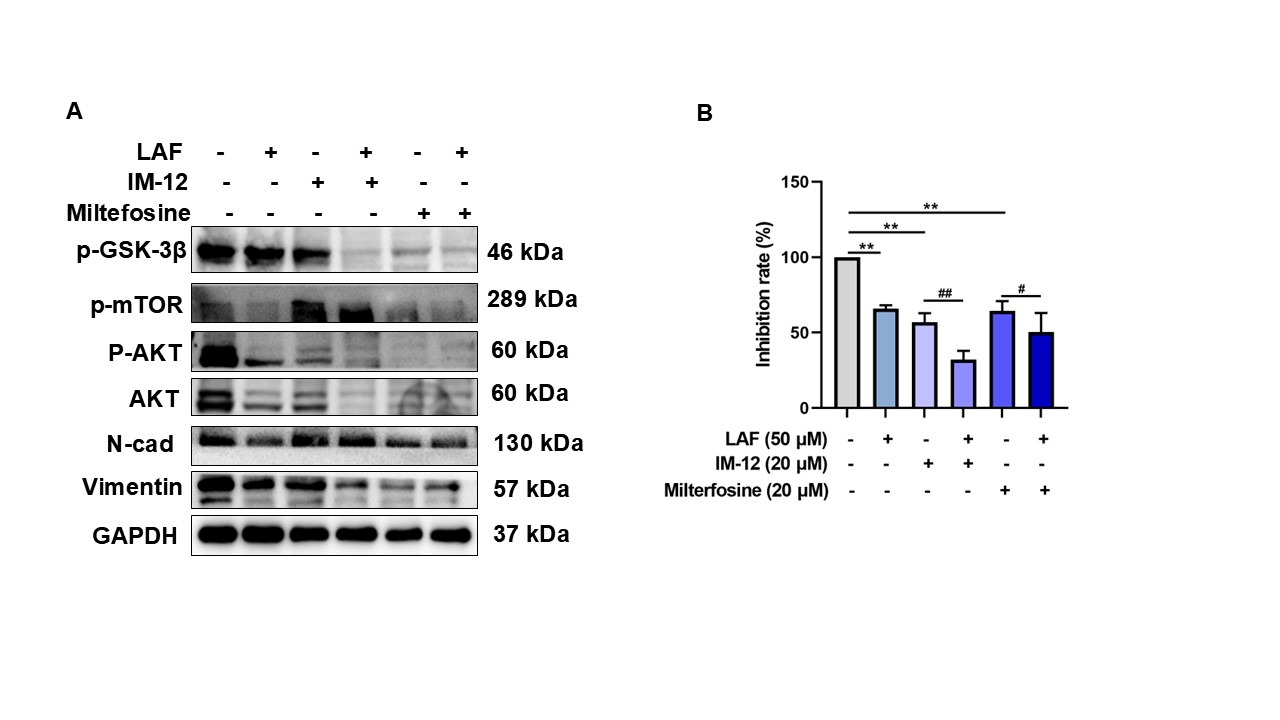

Supplement: Supplementary file 1 [file Image1.jpeg]
